# Sevoflurane Induces Coherent Slow-Delta Oscillations in Rats

**DOI:** 10.3389/fncir.2017.00036

**Published:** 2017-07-04

**Authors:** Jennifer A. Guidera, Norman E. Taylor, Justin T. Lee, Ksenia Y. Vlasov, JunZhu Pei, Emily P. Stephen, J. Patrick Mayo, Emery N. Brown, Ken Solt

**Affiliations:** ^1^Department of Anesthesia, Critical Care and Pain Medicine, Massachusetts General Hospital, BostonMA, United States; ^2^Department of Brain and Cognitive Sciences, Massachusetts Institute of Technology, CambridgeMA, United States; ^3^Department of Anaesthesia, Harvard Medical School, BostonMA, United States; ^4^Institute for Medical Engineering and Science, Massachusetts Institute of Technology, CambridgeMA, United States; ^5^Department of Neurobiology, Duke University, DurhamNC, United States; ^6^The Picower Institute for Learning and Memory, Massachusetts Institute of Technology, CambridgeMA, United States

**Keywords:** sevoflurane, rat, EEG, anesthesia, coherence

## Abstract

Although general anesthetics are routinely administered to surgical patients to induce loss of consciousness, the mechanisms underlying anesthetic-induced unconsciousness are not fully understood. In rats, we characterized changes in the extradural EEG and intracranial local field potentials (LFPs) within the prefrontal cortex (PFC), parietal cortex (PC), and central thalamus (CT) in response to progressively higher doses of the inhaled anesthetic sevoflurane. During induction with a low dose of sevoflurane, beta/low gamma (12–40 Hz) power increased in the frontal EEG and PFC, PC and CT LFPs, and PFC–CT and PFC–PFC LFP beta/low gamma coherence increased. Loss of movement (LOM) coincided with an abrupt decrease in beta/low gamma PFC–CT LFP coherence. Following LOM, cortically coherent slow-delta (0.1–4 Hz) oscillations were observed in the frontal EEG and PFC, PC and CT LFPs. At higher doses of sevoflurane sufficient to induce loss of the righting reflex, coherent slow-delta oscillations were dominant in the frontal EEG and PFC, PC and CT LFPs. Dynamics similar to those observed during induction were observed as animals emerged from sevoflurane anesthesia. We conclude that the rat is a useful animal model for sevoflurane-induced EEG oscillations in humans, and that coherent slow-delta oscillations are a correlate of sevoflurane-induced behavioral arrest and loss of righting in rats.

## Introduction

General anesthesia is a reversible, drug-induced state characterized by unconsciousness, amnesia, analgesia, and immobility in the setting of hemodynamic stability (Brown et al., 2010). Until recently, the study of anesthetic mechanisms has focused primarily on characterizing the actions of anesthetic drugs at molecular targets in the brain and spinal cord (Rudolph and Antkowiak, 2004; Hemmings et al., 2005; Franks, 2006). This important work established that multiple molecular targets are involved in general anesthesia (Eger et al., 1997; Grasshoff et al., 2005). However, it remains unclear how anesthetics produce profound changes in neurophysiology at the systems level to induce unconsciousness (Franks, 2008; Brown et al., 2011).

Sevoflurane is a halogenated ether anesthetic that is widely used in modern anesthesiology, due to favorable clinical characteristics such as rapid pharmacokinetics and lack of airway irritability. Recent studies using non-invasive measures such as electroencephalogram (EEG) and functional magnetic resonance imaging in humans have begun to characterize systems-level neurophysiological changes induced by sevoflurane (Gugino et al., 2001; Akeju et al., 2014; Blain-Moraes et al., 2015; Palanca et al., 2015; Kafashan et al., 2016). It has been reported that humans administered clinically relevant doses of sevoflurane exhibit slow-delta and alpha (8–12 Hz) oscillations in the scalp EEG (Akeju et al., 2014). Although anesthetic-induced oscillations may play a role in loss of consciousness (Lewis et al., 2012), their origins within the brain and precise relationship to unconsciousness are not known.

In order to assess the utility of the rat as an animal model for sevoflurane-induced EEG oscillations in humans, we characterized the EEG of rats receiving progressively higher doses of sevoflurane. We also characterized intracranial local field potentials (LFPs) within three brain regions thought to be involved in anesthetic-induced unconsciousness: the prefrontal cortex (PFC), parietal cortex (PC), and central thalamus (CT) (Ching et al., 2010; Ku et al., 2011; Mashour and Alkire, 2013; Baker et al., 2014).

## Materials and Methods

### Animal Care and Use

This study was carried out in accordance with the recommendations of the Guide for the Care and Use of Laboratory Animals, National Institutes of Health. Animal studies were approved by the Massachusetts General Hospital Institutional Animal Care and Use Committee. Nine adult male Sprague-Dawley rats (Charles River Laboratories, Wilmington, MA, United States) were housed on a standard day–night cycle (lights on from 7 am to 7 pm), and all experiments were conducted between 9 am and 5 pm. After habituation to the housing facility, animals were implanted with skull screws and intracranial electrodes for neural recordings, and at least 7 days were provided for recovery from surgery. In addition, a minimum of 3 days of rest was provided between experiments. Of note, the animals also had central venous catheters implanted for the administration of intravenous anesthetics. However, in this report we focus on results from sevoflurane administration.

### Pre-surgical Electrode Assembly

Three 3-cm pieces of insulated stainless steel wire (127 μm bare, 203.2 μm coated, no. 791400, A-M Systems) were cut. The insulation was removed from both ends of the wires with a flame torch, and one end of each wire was soldered to the female end of a male/female pin (363A, Plastics One). Two 4-cm 7-strand stainless steel wires (50.8 μm bare, 228.6 μm coated, no. 793500, A-M Systems) were cut for electromyography (EMG). The insulation was removed from both ends of the wires with a flame torch. One free end of each of the five resulting wires was fastened to a 32-channel electronic interface board (EIB) (EIB-36-PTB, Neuralynx) using small gold pins (EIB Small Pins, Neuralynx). Tetrodes were spun from insulated tungsten wire (tungsten 99.95% CS, 25.4 μm, California Fine Wire)^1^ on a custom electrode twister^2^. One to two tetrodes were secured within polyimide tubes (50.8 μm outer diameter, 406.4 μm inner diameter, High Performance Conductors) using cyanoacrylate glue (Thick, Great Planes), leaving 1–2 mm of electrode extending from the tube.

### Surgical Placement of EEG Screws and Intracranial Electrodes

**FIGURE 1:**
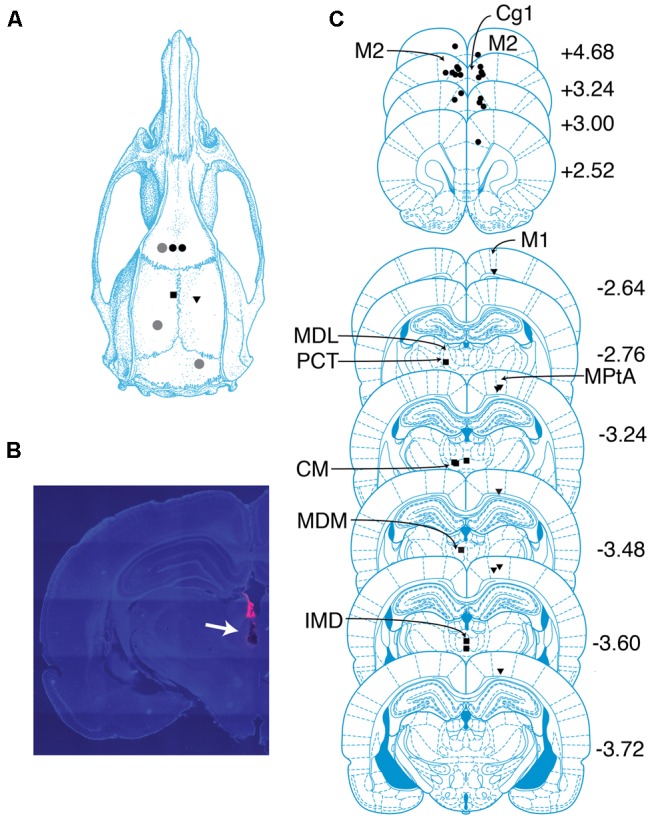
Overview of recording sites. **(A)** Schematic of targeted sites. Shapes represent locations where screws (gray circles) and electrodes (black circles, PFC; squares, CT; triangles, PC) were placed. **(B)** Coronal section containing the tract of an electrode localized to the CT. Tract is indicated with an arrow. Pink color is a dye used to aid with tract visualization. **(C)** Locations of probes in PFC, PC, and CT determined by histological analysis. Shapes same as in **(A)**. The center of each shape marks the tip of one electrode. Numbers indicate anterior–posterior position (mm) relative to bregma. cg1, cingulate cortex area 1; CM, central medial thalamic nucleus; IMD, intermediodorsal thalamic nucleus; M1, primary motor cortex; M2, secondary motor cortex; MDL, mediodorsal thalamic nucleus, lateral part; MDM, mediodorsal thalamic nucleus, medial part; MPtA, medial parietal association cortex; PCT, paracentral thalamic nucleus. Drawings in **(A)** and **(C)** are from Paxinos and Watson (2005).

Animals were rapidly anesthetized with isoflurane. Hair above the skull was removed with an electric razor. Animals were placed in a stereotaxic frame (Model 962, David Kopf Instruments, Tujunga, CA, United States) atop an electric heating pad and were administered 1.5–2.5% isoflurane for the remainder of the procedure. A rectal probe was inserted, and core temperature was continuously monitored and maintained within 37.5 ± 1°C. A pulse oximeter was attached to the paw for continuous monitoring of heart rate and blood oxygenation. Blunt ear bars were placed in the ear canals. Ophthalmic ointment was applied to the eyes every 1–2 h. Skin above the skull was cleaned with four rounds of betadine alternating with alcohol (70%). An incision was made from between the eyes to the rear of the skull, and blunt dissection was performed to expose the suture lines of the skull. Using a drill (Foredom, HP4-917) fastened to the stereotaxic frame, craniotomies were made for extradural frontal (3.00 mm anterior-posterior (AP), -2.50 mm medial-lateral (ML)) and rear (-6.00 mm AP, -4.50 mm ML) EEG and ground (-10.50 mm AP, 3.00 mm ML) screws and four to five anchor screws, and above targeted locations for electrodes within regions of the PFC, PC, and CT, including anterior cingulate cortex bilaterally (3.00 mm AP, ±0.80 mm ML, -2.00 mm dorsal-ventral (DV)), parietal association cortex (-3.60 mm AP, 2.00 mm ML, -2.00 to -1.00 mm DV), and central medial thalamus (-3.20 mm AP, -0.50 mm ML, -6.50 mm DV) (**Figure 1A**). Extradural teflon-insulated stainless steel screws attached to female pins (Plastics One, E363/20/SPC) were placed for EEG and ground connections, and stainless steel anchor screws (Stoelting, 51457) were placed. Electrodes were individually fastened to the arm of the stereotaxic frame by melting poly(ethylene glycol) (PEG; Sigma Aldrich, 309028-250g) with a soldering iron on low heat, and applying the melted PEG to a guide tube containing electrodes lying flat on the stereotax arm. Prior to lowering electrodes, the dura above the target site was removed with a 30-gauge needle, and electrodes were dipped in an organic dye, 1,1′-dioctadecyl-3,3,3′,3′-tetramethylindocarbocyanine perchlorate (diI) (Vybrant DiI Cell-Labeling Solution, Thermo Fisher Scientific), to aid with tract visualization during post-mortem histological analysis (**Figure 1B**). Once lowered, guide tubes containing electrodes were secured to the skull and nearby screws with dental acrylic (Teets methyl methacrylate and dental cement liquid), then removed from the arm of the stereotaxic frame by liquefying the PEG connection with heat or saline. Electrodes were fastened to the EIB using small gold pins (EIB Small Pins, Neuralynx). Blunt dissection was performed above the trapezius muscles and nuchal EMG wires fastened to the EIB were placed. EEG and ground screws were connected to the EIB by mating male/female pins. The implant was encased with dental acrylic. Skin was closed with absorbable suture. Triple antibiotic ointment was applied around the implant. During surgery, animals received 1–2 mL saline subcutaneously every 2–3 h to maintain hydration. Following surgery, animals received 4 mg/kg ketoprofen (Zoetis) subcutaneously for pain relief the day of and 2 days post-surgery. Animals were given at least 7 days to recover before undergoing experiments.

### EEG and LFP Recordings

Recordings took place in a custom-built acrylic chamber (30.48 cm × 30.48 cm × 30.48 cm) with separate ports for wires, gas inflow, gas scavenging, and monitoring of anesthetic agents. Experiments began with animals awake in a box pre-flushed with oxygen. Sevoflurane (Henry Schein Animal Health) in oxygen was administered in seven progressively higher doses: 1.6, 1.8, 2.0, 2.2, 2.4, 2.6, and 2.8%. To rapidly transition to higher doses, 8% sevoflurane was briefly administered at 10 L/min. Targeted doses were held for 30–35 min with 1 L/min fresh gas flow, and the sevoflurane concentration was continuously monitored with an Ohmeda 5250 anesthetic gas analyzer (GE Healthcare) to ensure a steady state. The first 20 min at each dose were allowed for equilibration of the animal to the chamber concentration. Within minutes of the onset of frontal slow-delta oscillations, the rats were placed in the lateral decubitus position on a non-electric heating pad (20.32 cm × 20.32 cm, Deltaphase Isothermal Pad, Braintree Scientific) that had been warmed to 38–40°C in a water bath. Once animals were flaccid and unreactive upon handling, a rectal probe was inserted, and core temperature was continuously monitored. The heating pad was replaced as needed in order to maintain temperature within 37.5 ± 1°C. At the end of the final sevoflurane dose, the box remained closed and was flushed with 100% oxygen at 10 L/min, leading to a steady decrease in sevoflurane concentration over approximately 10 min.

Recordings began about 15 min prior to the start of sevoflurane administration and ended after animals regained movement. Signals were continuously recorded with an Omniplex D Neural Data Acquisition System. Analog signals were amplified with a 20× gain 32-channel headstage (HST/32V-G20 LN, Plexon), digitized with a sampling rate of 40 kHz with a Plexon MiniDigiAmp, and digitally filtered (Bessel, 4 poles, 200 Hz cutoff) and downsampled to 1 kHz in OmniPlex Server. In some experiments, a 32-channel commutator (COM/32, Plexon) was used. Video was recorded continuously (Stingray, Allied Vision), and the *X*–*Y* coordinates of 1–2 light emitting diodes attached to the headstage were sampled at 30 Hz (CinePlex Tracking software, Plexon).

### Behavioral Endpoints

We assessed loss and return of the righting reflex (LOR and ROR), which are commonly used behavioral endpoints for loss and return of consciousness in rodents. We defined LOR as failure of the animal to return all four paws to the ground following placement in the lateral decubitus position. In order to observe the neurophysiological and behavioral time course of induction with sevoflurane in the absence of external stimulation, we did not test for LOR continuously, but only when placing animals on heating pads as described above. We defined ROR as all four paws touching the ground following termination of sevoflurane administration.

We also defined endpoints for behavioral arrest and return of behavior. We defined loss of movement (LOM) as LED(s) attached to the headstage becoming motionless and remaining so for at least 70 s during induction with 1.6% sevoflurane. We defined return of movement (ROM) as the return of LED movement following the last dose of sevoflurane. Video and motion tracking data were used to determine the precise times when ROR, LOM, and ROM occurred.

### Histological Analysis

Electrode placement was determined via post-mortem histological analysis. Animals were deeply anesthetized with a high dose of isoflurane (5%) until cessation of breathing occurred, then perfused with phosphate-buffered saline (1X) followed by formalin (10%). Brains were stored in formalin for at least 24 h, after which 60 μm coronal sections were cut using a vibratome (VT 1200S, Leica Microsystems). Sections were mounted on slides with a medium containing 4′,6-diamidino-2-phenylindole (H-1200, Vector Laboratories) and imaged with an Axio Imager M2 fluorescence microscope (Zeiss). Images were overlaid with best-fitting sections from the *The Rat Brain in Stereotaxic Coordinates* (Paxinos and Watson, 2005), and the ends of tracts were used to determine probe locations.

### Data Analysis

Data analysis was performed in MATLAB R2015b (MathWorks). Spectral and coherence calculations were implemented with functions from the Chronux toolbox ^3^ (Mitra and Bokil, 2008).

#### Selection of Time Periods for Group-Level Analyses

We selected time periods for group-level analyses with the goal of characterizing dynamics observed in all animals during induction, maintenance, emergence, and recovery from sevoflurane anesthesia. For each animal, an awake baseline was selected as a 1-min period before sevoflurane administration during which the animal was relatively motionless, but did not exhibit spindles or high-amplitude slow-delta oscillations in the frontal EEG suggestive of non-rapid eye movement sleep. The 4-min period beginning 1 min before the start of sevoflurane administration was selected for analysis of the frontal EEG during the first minutes of induction. For analysis of LFPs during early induction, the 1-min period beginning 2 min after the start of sevoflurane administration was selected. In two animals, this period was shifted either backward or forward by about 30 s to avoid motion artifacts. The 2-min period beginning 1 min before LOM was selected for analysis of the frontal EEG around the time of LOM. For analysis of LFPs around the time of LOM, 30-s periods beginning 30 s before and after LOM were selected. The 1-min period beginning 28 min after the start of 2.2% sevoflurane administration was selected for analysis of the frontal EEG and LFPs at a dose of sevoflurane sufficient to maintain LOR. In order to enable characterization of slow-delta oscillations, which was by far the most common dynamic observed following LOR, in two animals, the 2.2% period was shifted earlier but still after the 20-min equilibration period in order to exclude theta (4–8 Hz) oscillations or burst suppression. Around the time of ROM, animals exhibited variable frontal dynamics, complicating a group analysis around the time of ROM. However, all animals exhibited a decrease in slow-delta power either before or at ROM. For analysis of the frontal EEG and LFPs during the last epoch of elevated slow-delta power, the 1 min before slow-delta power returned to baseline levels was selected using a threshold procedure described below. The first artifact-free minute after ROR was selected for analysis of the frontal EEG and LFPs during recovery.

#### Analysis of EEG and EMG Signals

Spectrograms of the frontal EEG were calculated using the multitaper method. The Chronux function “mtspecgramc” was used with a 2-s sliding window incremented by 0.05 s, a half-bandwidth of 1 Hz, and three tapers. Power values were converted to decibels. Group-level median difference spectrograms were derived from spectrograms for individuals during the awake baseline period and periods of interest. Within animals, the median power during the 1-min awake baseline period was subtracted from power during periods of interest, yielding difference spectrograms for individuals. Group-level median difference spectrograms display the median power difference value at every time-frequency point across subjects.

EMG power for individual animals was calculated by passing signals via the MATLAB function “filtfilt” through a 200th order finite impulse response (FIR) 80–200 Hz bandpass filter constructed with the MATLAB function “fir1,” and squaring the resulting values. Within animals, EMG power was expressed as a percentage of the median EMG power during awake baseline. Group-level EMG power reflects the median of these percentages.

#### Analysis of LFPs

Only signals from probes localized to PFC, PC, or CT were included in group-level analyses of LFPs. Each animal contributed at most a single signal per targeted location (PFC right hemisphere, PFC left hemisphere, PC right hemisphere, and CT left hemisphere).

##### Spectral calculations

Power spectra of PFC, PC, and CT LFPs were generated using the multitaper method. The Chronux function “mtspectrumc” was used with a half-bandwidth of 1 Hz and five tapers. For each animal, awake baseline periods and periods of interest were divided into 3-s trials, and the power for each trial was calculated and converted to decibels. The power during the awake baseline period was subtracted from power during the period of interest for each trial. The median power difference across trials pooled from all animals was calculated. A bootstrap procedure was performed in order to determine the 95% confidence intervals of the median power difference. At each frequency, power difference values were randomly sampled with replacement a number of times equal to the number of trials, and the median power difference was calculated. This process was repeated 1000 times, and the 2.5th and 97.5th percentiles of the resulting distribution were identified. As a half-bandwidth of 1 Hz implies a frequency resolution of 2 Hz, we considered power during a period of interest to be significantly greater than power during awake baseline if the 2.5th percentile of the power difference was greater than 0 for a contiguous set of frequencies spanning at least 2 Hz. PFC LFPs from both the left and right hemispheres were included in the calculation of power difference spectra for the PFC LFP.

Spectrograms of PFC LFPs were generated in the same manner as described above for EEG signals.

##### Coherence and phase calculations

For signals *x* and *y*,

$$\begin{array}{c} \mathrm{Coherency}=\frac{S_{xy}}{\sqrt{S_{xx}S_{yy}}} \end{array}$$

where *S_xy_* is the cross-spectral density of *x* and *y, S_xx_* is the autospectral density of *x* and *S_yy_* is the autospectral density of *y*. The magnitude of coherency gives coherence, a measure of the degree of synchrony between two signals. The angle of coherency gives the phase relationship between two signals.

Coherency was calculated for pairs of LFPs: PFC–CT (ipsilateral), PC–CT (contralateral), PFC–PFC (contralateral), and PFC–PC (ipsilateral). The awake baseline period and periods of interest were divided into 3-s trials. Cross-spectral density and autospectral density values for each trial were calculated using the Chronux function “coherencyc” with a half-bandwidth of 1 Hz and five tapers. The mean cross-spectral and auto-spectral density values for trials pooled across animals were related by the formula for coherency. The magnitude of coherency yielded “trial-averaged coherence” (Cohen, 2014). The phase angle of coherency was calculated and expressed in degrees and as a time delay. Trial-averaged coherence values for the awake baseline were subtracted from those for periods of interest, yielding coherence difference. A bootstrap procedure was performed to determine the 95% confidence intervals for trial-averaged coherence, coherence difference, and phase angle. At each frequency, cross-spectral and autospectral values were randomly sampled with replacement a number of times equal to the number of trials. Trial-averaged coherence, coherence difference, and phase angle were then calculated in the same manner as above. This process was repeated 1000 times, and the 2.5th and 97.5th percentiles of the resulting distributions were identified. Coherence during a period of interest was considered significantly greater than coherence during awake baseline if the 2.5th percentile of the coherence difference was greater than 0 for a contiguous set of frequencies spanning at least 2 Hz.

Coherograms were generated using the Chronux function “cohgramc”. A 2-s sliding window incremented by 0.05 s, a half-bandwidth of 1 Hz, and three tapers were used. Within animals, the median coherence during the 1-min awake baseline period was subtracted from coherence values during periods of interest, yielding difference coherograms for individuals. Group-level median difference coherograms display the median coherence difference value at every time-frequency point across individuals.

Filtered LFPs were generated to illustrate the group-level phase relationship between two signals in a frequency range of interest, most often the range in which coherence between the signals significantly increased during a period of interest from awake baseline. LFPs were passed via the MATLAB function “filtfilt” through a 200th order FIR bandpass filter constructed with the MATLAB function “fir1”.

##### Determination of the last period of elevated slow-delta power

For each animal, the time at which slow-delta power in the PFC LFP in the right hemisphere returned to baseline levels was determined using a threshold procedure. The MATLAB function “fir1” was used to produce a 200th order FIR 0.1–4 Hz bandpass filter. The right PFC LFP during the awake baseline period and the 10-min period following the end of sevoflurane administration was passed through this filter using the MATLAB function “filtfilt.” These values were squared, then smoothed with a 5000-point (5-s) moving average using the MATLAB function “smooth.” The first time following the end of sevoflurane administration at which slow-delta power was equal to or less than the mean plus two standard deviations of the slow-delta power during the awake baseline period was determined, excluding periods of burst suppression which were present in some animals up to about 1 min following the end of sevoflurane administration. The 1 min preceding this time was considered the last minute of elevated slow-delta power.

##### Frequency band definitions

Frequency bands were defined as follows: slow-delta (0.1–4 Hz), theta (4–8 Hz), alpha (8–12 Hz), beta (12–25 Hz), low beta (12–15 Hz), high beta (20–25 Hz), and low gamma (25–40 Hz).

## Results

### Probe Locations

Eighteen probes were localized to regions within bilateral PFC, including three to secondary motor cortex and 15 to anterior cingulate cortex (**Figure 1C**). Probes in the right hemisphere spanned (2.5–4.7 mm AP, 0.7–1.0 mm ML, and -2.7 to -1.5 mm DV), and probes in the left hemisphere spanned (3.0–4.7 mm AP, -1.5 to -0.4 mm ML, and -2.2 to -1.4 mm DV). Seven probes were localized to regions within PC, including one to primary motor cortex, one to the border between primary motor cortex and parietal association cortex, and five to parietal association cortex (**Figure 1C**). Probes in PC spanned (-3.7 to -2.6 mm AP, 1.8–2.2 mm ML, and -1.8 to -1.0 mm DV). Adopting Schiff’s terminology (Schiff, 2008), we refer to intralaminar and related midline nuclei as CT. Seven probes were localized to CT, including one to the border between the lateral mediodorsal thalamic nucleus and the paracentral nucleus, one to the median mediodorsal thalamic nucleus, one to the intermediodorsal thalamic nucleus, and four to the central medial thalamic nucleus (**Figure 1C**). Probes in CT spanned (-3.6 to -2.8 mm AP, -1.4–0 mm ML, and -6.4 to -5.6 mm DV).

### Overview of Oscillatory Dynamics Observed

**FIGURE 2:**
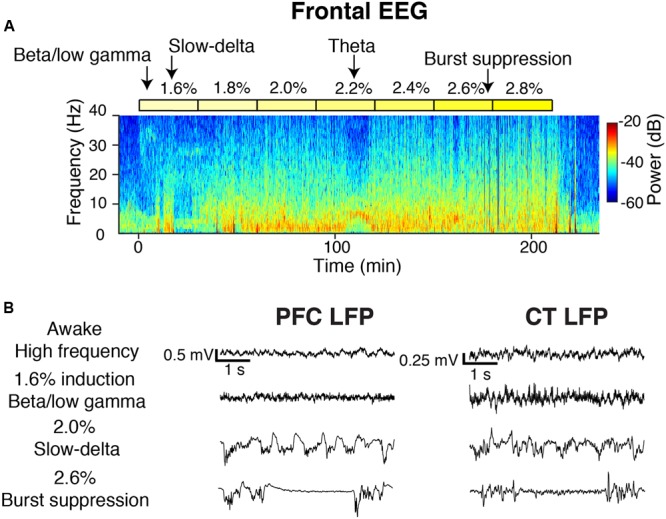
Overview of oscillatory dynamics observed during the 1.6–2.8% sevoflurane ramp. **(A)** Spectrogram of the frontal EEG from a representative animal during the sevoflurane ramp. Yellow blocks mark periods during which sevoflurane was set to the indicated dose. Arrows point to oscillatory dynamics. From this animal, **(B)** PFC (left) and CT (right) LFPs from four 5-s periods.

During induction with 1.6% sevoflurane, animals exhibited increased beta/low gamma power in the frontal EEG (**Figure 2A**). Increased slow-delta power was the most frequently observed frontal EEG dynamic during 1.6–2.8% sevoflurane administration (**Figure 2A**). Within a range of higher doses (2.2–2.8%), all animals exhibited burst suppression, characterized by alternating periods of high frequency activity and isoelectricity (**Figure 2A**). Three animals exhibited brief periods (1–10 min) of increased theta or alpha power and decreased slow-delta power at a range of doses (1.8–2.8%) (**Figure 2A**). Similar dynamics were observed in LFPs (**Figure 2B**).

### Beta/Low Gamma Dynamics during Induction

**FIGURE 3:**
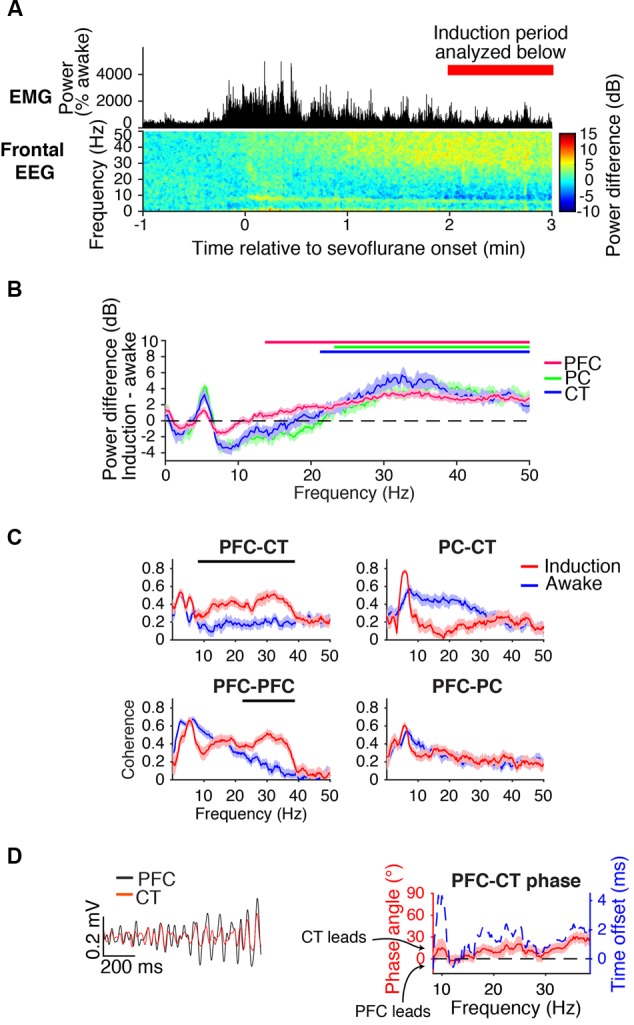
Dynamics during induction. **(A)** Group EMG power and group difference spectrogram of the frontal EEG aligned to the start of sevoflurane administration. Red rectangle with text marks the period analyzed in **(B–D)**. **(B)** Group power difference for PFC (red), PC (green), and CT (blue) LFPs. Shading indicates 95% confidence intervals, and red, green, and blue horizontal lines mark ranges of significant power increase from awake baseline for PFC, PC, and CT LFPs, respectively. **(C)** Group coherence between LFPs during the 1.6% sevoflurane induction (red) and awake (blue) states. Shading indicates 95% confidence intervals, and horizontal lines mark ranges of significant coherence increase from awake baseline. **(D)** Left: PFC (black) and CT (red) LFPs from a representative animal filtered in the range of significant PFC–CT LFP coherence increase shown in **(C)**. Right: group PFC–CT LFP phase relationship in the range of significant PFC–CT LFP coherence increase shown in **(C)**, in terms of phase angle (left *y*-axis, red line) and time offset (right *y*-axis, blue dotted line). Shading indicates 95% confidence intervals for phase angle. A positive phase relationship indicates that the first signal lags the second at the indicated frequency.

During the first minutes of sevoflurane administration, animals became highly active, resulting in increased EMG power and a theta oscillation characteristic of moving rodents (Vanderwolf, 1969; **Figure 3A**). Beta/low gamma power increased in the frontal EEG (**Figure 3A**) and PFC, PC, and CT LFPs (**Figure 3B**).

Beta/low gamma coherence increased from baseline between PFC and CT LFPs and between bilateral PFC LFPs (**Figure 3C**). In the range of significant PFC–CT coherence increase shown in **Figure 3C**, the PFC LFP had a positive phase relationship with the CT LFP, indicating that in this frequency range, the CT LFP led the PFC LFP (**Figure 3D**).

### A Transition from Beta/Low Gamma to Slow-Delta Dynamics Correlated with Behavioral Arrest

**FIGURE 4:**
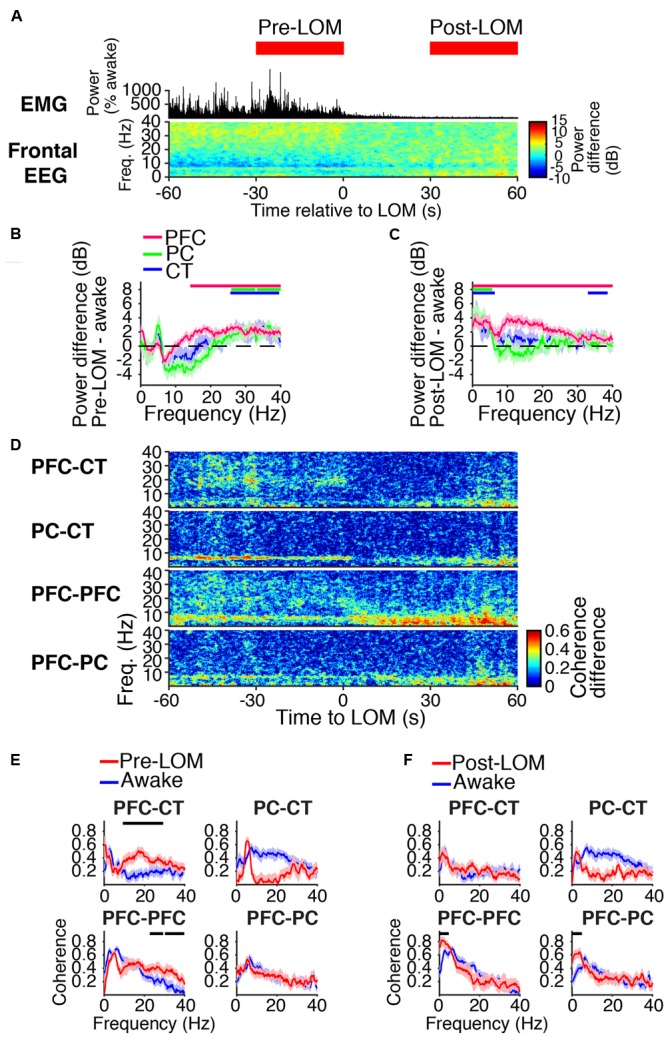
Dynamics around the time of loss of movement. **(A)** Group EMG power and group difference spectrogram of the frontal EEG aligned to LOM. Red rectangles mark the periods analyzed in **(B,C,E,F)**. **(B,C)** Group power difference between 30-s periods immediately before **(B)** or 30 s after **(C)** LOM and awake baseline for PFC (red), PC (green), and CT (blue) LFPs. Shading indicates 95% confidence intervals. Red, green, and blue horizontal lines mark ranges of significant power increase relative to awake baseline for PFC, PC, and CT LFPs, respectively. **(D)** Group difference coherograms for pairs of LFPs aligned to LOM. **(E,F)** Group coherence during awake baseline (blue) and periods before **(E)** or after **(F)** LOM (red). Shading indicates 95% confidence intervals, and horizontal lines mark ranges of significant coherence increase from awake baseline.

During induction with 1.6% sevoflurane, animals eventually transitioned from a state of high motor activity to motionlessness. The median time to LOM after the start of sevoflurane administration was 5 min and 8 s [interquartile range (IQR): 2 min and 42 s]. LOM coincided with a decrease in muscle tone and beta/low gamma power in the frontal EEG (**Figure 4A**). Following LOM, slow-delta power increased in the frontal EEG (**Figure 4A**). Similar power changes occurred in the PFC, PC, and CT LFPs. Prior to LOM, beta/low gamma power remained elevated from awake baseline in PFC, PC, and CT LFPs (**Figure 4B**). Following LOM, slow-delta power increased in PFC, PC, and CT LFPs, and power increased in a broad range in the PFC LFP (**Figure 4C**).

Before LOM, beta/low gamma coherence was elevated from awake baseline between PFC and CT LFPs and between bilateral PFC LFPs (**Figures 4D,E**). An abrupt decrease in PFC–CT beta/low gamma coherence coincided with LOM (**Figure 4D**). Following LOM, slow-delta coherence increased between bilateral PFC LFPs and between PFC and PC LFPs (**Figures 4D,F**).

### Coherent Slow-Delta Oscillations at Higher Doses of Sevoflurane

**FIGURE 5:**
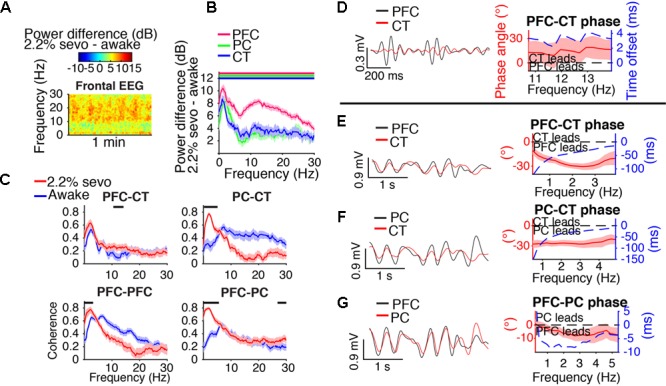
Slow-delta dynamics during 2.2% sevoflurane. **(A)** Group difference spectrogram of the frontal EEG during slow-delta dominated periods at 2.2% sevoflurane. The same period is analyzed in **(B–G)**. **(B)** Group power difference between 2.2% sevoflurane and awake baseline for PFC (red), PC (green), and CT (blue) LFPs. Shading indicates 95% confidence intervals. Red, green, and blue horizontal lines mark ranges of significant power increase relative to awake baseline for PFC, PC, and CT LFPs, respectively. **(C)** Group coherence during the awake (blue) and 2.2% sevoflurane (red) states for pairs of LFPs. Shading indicates 95% confidence intervals, and horizontal lines mark ranges of significant coherence increase from awake baseline. **(D–G)** Left: LFPs from a representative animal filtered in the frequency range shown in the corresponding plot on the right. Right: Group phase relationship between two LFPs shown in terms of phase angle (left *y*-axis, red line) and time offset (right *y*-axis, blue dotted line). A positive phase relationship indicates that the first signal lags the second, and a negative phase relationship indicates that the first signal leads the second. **(D)** Group PFC–CT LFP phase relationship in the range of significant PFC–CT LFP coherence increase shown in **(C)**. **(E)** Group PFC–CT LFP phase relationship in the slow-delta range (note: PFC–CT coherence did not significantly increase in this range). **(F)** Group PC–CT LFP relationship in the range of significant PC–CT LFP coherence increase shown in **(C)**. **(G)** Group PFC–PC LFP phase relationship in the range of significant PFC–PC LFP coherence increase within the slow-delta range shown in **(C)**.

Spectral and coherence analyses returned similar results for periods dominated by slow-delta oscillations for a range of doses sufficient to maintain LOR. Results for a representative dose, 2.2% sevoflurane, are shown in **Figure 5**. During slow-delta dominated periods at 2.2% sevoflurane, frontal EEG power increased in a broad range, with peak increases in the slow-delta and higher frequency (alpha/beta/low gamma) ranges (**Figure 5A**). Power similarly increased in a broad range in LFPs, with peak increases in the slow-delta and alpha/beta ranges (**Figure 5B**). These power increases were greatest in magnitude in the PFC LFP (**Figure 5B**). For LFPs of all sites, increases in power were greatest in the slow-delta range (**Figure 5B**).

Slow-delta coherence increased between PC and CT LFPs, between bilateral PFC LFPs, and between PFC and PC LFPs (**Figure 5C**). Alpha/low beta coherence increased between PFC and CT LFPs (**Figure 5C**). In the range of significant PFC–CT LFP coherence increase shown in **Figure 5C**, the PFC LFP had a positive phase relationship with the CT LFP, indicating that in this frequency range, the CT LFP led the PFC LFP (**Figure 5D**). In the slow-delta range, in which PFC–CT LFP coherence increased though not significantly from awake baseline, the PFC LFP had a negative relationship with the CT LFP, indicating that in this frequency range, the PFC LFP led the CT LFP (**Figure 5E**). In the range of significant PC–CT LFP coherence increase shown in **Figure 5C**, the PC LFP had a negative phase relationship with the CT LFP, indicating that in this frequency range, the PC LFP led the CT LFP (**Figure 5F**). In the range of significant PFC–PC LFP coherence increase within the slow-delta range shown in **Figure 5C**, the PFC LFP had a negative phase relationship with the PC LFP, indicating that in this frequency range, the PFC LFP led the PC LFP (**Figure 5G**).

### Animals Took Distinct Paths during Emergence

**FIGURE 6:**
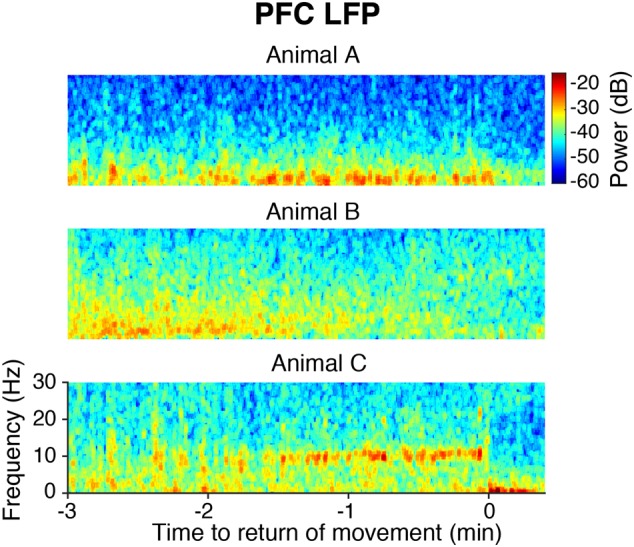
Spectrograms of the PFC LFP from three animals aligned to ROM.

Following the end of sevoflurane administration and inflow of 10 L/min oxygen, the median time to ROM was 6 min and 17 s [IQR: 2 min and 30 s], and the median time to ROR was 6 min and 23 s [IQR: 2 min and 15 s]. In the PFC LFPs of all animals, slow-delta power returned to awake baseline levels before or at the time of ROM. However, emergence dynamics varied among rats, as illustrated in **Figure 6**. In animal A, PFC LFP slow-delta power abruptly decreased at the time of ROM, whereas in animal B, PFC LFP slow-delta power gradually decreased prior to ROM. Animal C exhibited an alpha oscillation in the PFC LFP during emergence that was not observed during induction.

### During Emergence, Cortico-Cortical Slow-Delta Coherence Remained Elevated

**FIGURE 7:**
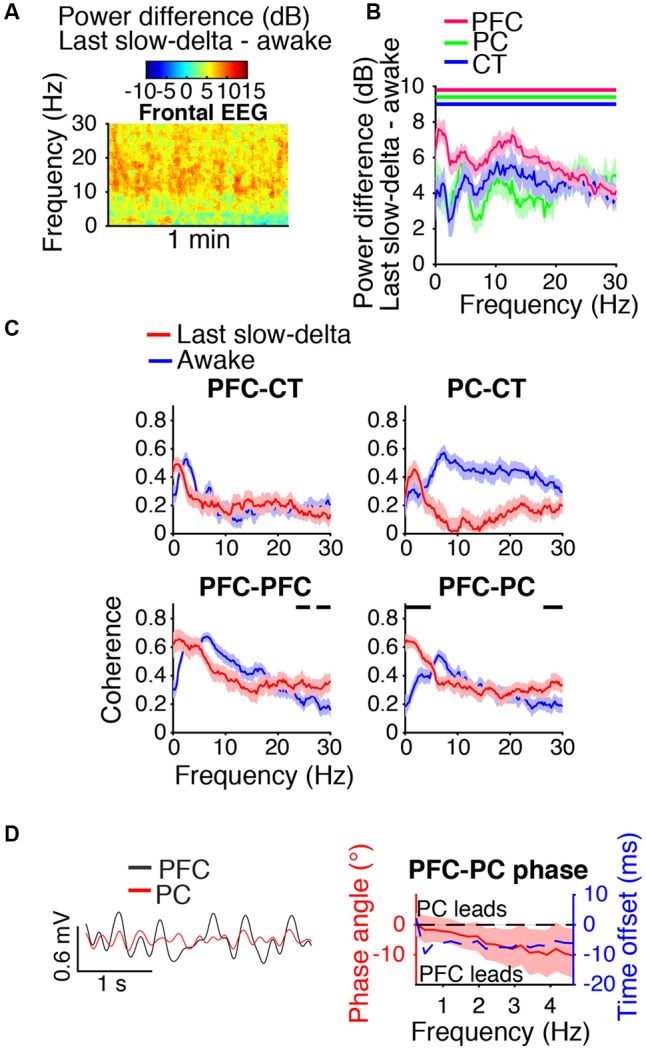
Dynamics during the last minute of elevated slow-delta power. **(A)** Group difference spectrogram of the frontal EEG during the last minute of elevated slow-delta power. The same period is analyzed in **(B–D)**. **(B)** Group power difference for PFC (red), PC (green), and CT (blue) LFPs. Shading indicates 95% confidence intervals. Red, green, and blue horizontal lines mark ranges of significant power increase relative to awake baseline for PFC, PC, and CT LFPs, respectively. **(C)** Group coherence between LFPs during the awake baseline (blue) and last minute of elevated slow-delta power (red) states. Shading indicates 95% confidence intervals, and horizontal lines mark ranges of significant coherence increase from awake baseline. **(D)** Left: PFC (black) and PC (red) LFPs from a representative animal filtered in the range of significant PFC–PC LFP coherence increase within the slow-delta range shown in **(C)**. Right: Group PFC–PC LFP phase relationship shown for the range of significant PFC–PC LFP coherence increase shown in **(C)**, in terms of phase angle (left *y*-axis, red line) and time offset (right *y*-axis, blue dotted line). Shading indicates 95% confidence intervals for phase angle. A negative phase relationship indicates that the first signal leads the second.

During the last minute of elevated slow-delta power (**Figure 7A**), slow-delta power in CT and PC LFPs was closer to awake baseline levels than in the PFC LFP (**Figure 7B**). Slow-delta coherence between bilateral PFC LFPs and between PC and CT LFPs had returned to baseline levels, despite remaining elevated between PFC and PC LFPs (**Figure 7C**). In the range of significant PFC–PC LFP coherence increase shown in **Figure 7C**, the PFC LFP had a negative phase relationship with the PC LFP, indicating that in this frequency range, the PFC LFP led the PC LFP (**Figure 7D**).

### Beta/Low Gamma Power was Elevated during Recovery

**FIGURE 8:**
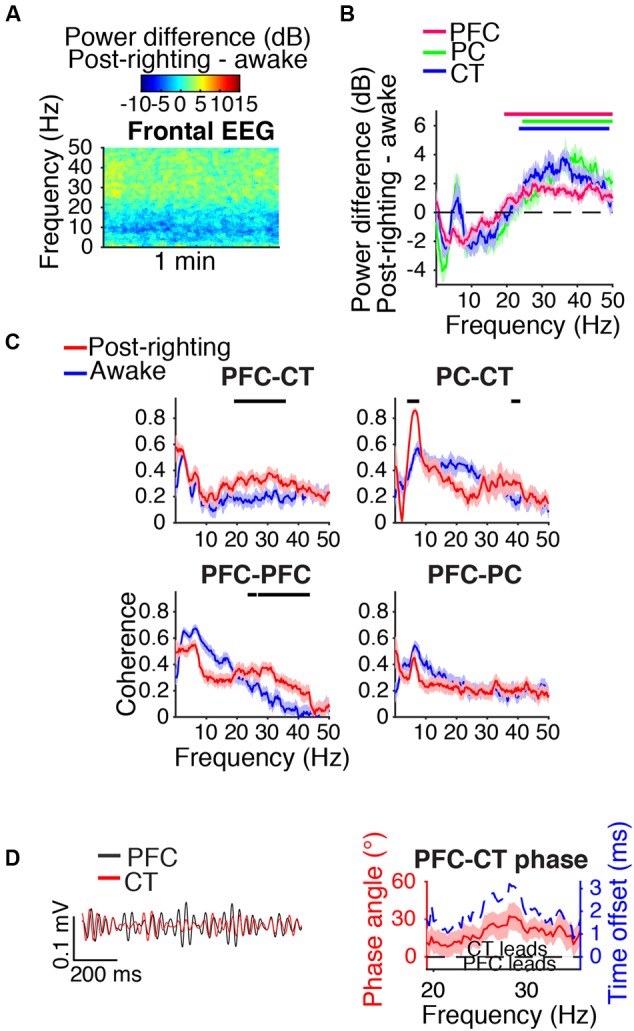
Dynamics following ROR. **(A)** Group difference spectrogram of the frontal EEG during the first artifact-free minute following righting. The same period is analyzed in **(B–D)**. **(B)** Group power difference for PFC (red), PC (green), and CT (blue) LFPs. Shading indicates 95% confidence intervals. Red, green, and blue horizontal lines mark ranges of significant power increase relative to awake baseline for PFC, PC, and CT LFPs, respectively. **(C)** Group coherence between LFPs during awake baseline (blue) and post-righting (red) states. Shading indicates 95% confidence intervals, and horizontal lines mark ranges of significant coherence increase from awake baseline. **(D)** Left: PFC (black) and CT (red) LFPs from a representative animal filtered in the range of significant PFC–CT LFP coherence increase shown in **(C)**. Right: Group PFC–CT LFP phase relationship shown for the range of significant PFC–CT LFP coherence increase shown in **(C)**, in terms of phase angle (left *y*-axis, red line) and time offset (right *y*-axis, blue dotted line). Shading indicates 95% confidence intervals for phase angle. A positive phase relationship indicates that the first signal lags the second.

During the first artifact-free minute following righting, beta/low gamma power was elevated relative to awake baseline levels in the frontal EEG (**Figure 8A**) and PFC, PC, and CT LFPs (**Figure 8B**). Beta/low gamma coherence between PFC and CT LFPs and between bilateral PFC LFPs was elevated (**Figure 8C**). In the range of significant PFC–CT LFP coherence increase shown in **Figure 8C**, the PFC LFP had a positive phase relationship with the CT LFP, indicating that in this frequency range, the CT LFP led the PFC LFP (**Figure 8D**).

## Discussion

In this study, extradural EEG and intracranial LFPs in the PFC, PC, and CT were recorded in rats under sevoflurane anesthesia. At low doses, sevoflurane increased beta/low gamma power in the frontal EEG and PFC, PC and CT LFPs, and increased PFC–CT and bilateral PFC–PFC LFP beta/low gamma coherence. LOM correlated with an abrupt decrease in PFC–CT LFP beta/low gamma coherence, and was followed by increased slow-delta power in PFC, PC, and CT LFPs and increased cortico-cortical slow-delta coherence. At higher doses, sevoflurane further increased slow-delta power in the frontal EEG and PFC, PC and CT LFPs and increased PC–CT, bilateral PFC–PFC, and PFC–PC LFP slow-delta coherence. During emergence, slow-delta power decreased in the frontal EEG and PFC, PC and CT LFPs. During the last minute of elevated slow-delta power, PFC–PC LFP slow-delta coherence remained elevated, while PC–CT and bilateral PFC–PFC LFP slow-delta coherence had returned to baseline levels. During recovery, beta/low gamma power was elevated in the frontal EEG and PFC, PC and CT LFPs, and beta/low gamma coherence was elevated between PFC–CT and bilateral PFC–PFC LFPs.

### The Rat as an Animal Model for Sevoflurane-Induced EEG Oscillations in Humans

In humans, low doses of sevoflurane increase beta/low gamma power in the frontal EEG (Gugino et al., 2001; Brown et al., 2010), and higher doses increase slow-delta and alpha power (Akeju et al., 2014; Purdon et al., 2015) and induce burst suppression (Pilge et al., 2014). Loss of consciousness in humans under sevoflurane and propofol anesthesia is associated with an increase in slow-delta power in the frontal EEG (Gugino et al., 2001; Purdon et al., 2013). In rats administered sevoflurane, we observed similar frontal EEG dynamics. Increased slow-delta power and burst suppression have also been previously observed in isoflurane-anesthetized rats (Silva et al., 2010; Hudson et al., 2014).

Despite these similarities in sevoflurane-induced dynamics, burst suppression appears to occur at a lower dose in rats than in humans. In humans, burst suppression occurs at roughly 1.4 times the minimum alveolar concentration (MAC), the dose required to prevent a motor response to a surgical stimulus in half of subjects. Rats in this study exhibited burst suppression at 2.2–2.8% sevoflurane, or roughly 0.9–1.1 MAC (Kashimoto et al., 2006). In addition, sevoflurane-induced alpha dynamics may differ between the two species. In rats, frontal EEG alpha power increased under sevoflurane anesthesia, but beta and low gamma power increased as well. In contrast, increases in higher frequency power may be more localized to the alpha band in humans (Akeju et al., 2014). Anatomical differences may underlie these apparent dissimilarities in sevoflurane-induced dynamics in rats and humans.

### In Rats, Sevoflurane-Induced Paradoxical Excitation Extends Beyond the Cortex

During the first minutes of sevoflurane administration, rats became active and beta/low gamma power increased in the frontal EEG and PFC, PC and CT LFPs. In humans, such counterintuitive behavioral and electrophysiological signs of arousal following administration of a general anesthetic is termed paradoxical excitation (Brown et al., 2010). Propofol and sevoflurane, which both potentiate γ-aminobutyric acid type A (GABA_A_) receptors, are known to induce paradoxical excitation at low doses in humans (Gugino et al., 2001; Brown et al., 2010). Although sevoflurane has additional molecular targets that may be important for its anesthetic actions (Eckenhoff, 2001), it is possible that both sevoflurane and propofol induce paradoxical excitation in mechanistically similar ways involving GABA_A_ receptors. Entertaining this possibility, we interpret our results in the context of a model for beta oscillation generation during propofol-induced paradoxical excitation (McCarthy et al., 2008). According to this model, binding of propofol to GABA_A_ receptors of cortical inhibitory interneurons is sufficient to shift these neurons from synchronous to asynchronous firing, which in turn results in the network-level generation of beta oscillations. Our results are consistent with the possibility that beta oscillations induced by a low dose of a GABA_A_ receptor enhancer are cortically generated, as low-dose sevoflurane increased beta power in cortical LFPs. However, our findings also raise the possibility that a subcortical structure, the CT, is engaged in oscillatory activity during paradoxical excitation induced by a low dose of a GABA_A_ receptor enhancer. High beta power increased in the CT LFP, as was previously observed in the central medial thalamus of rats administered propofol (Baker et al., 2014). We additionally found that beta coherence increased between CT and PFC LFPs. Further work is needed to clarify the roles of thalamus and cortex in beta oscillation generation and maintenance during anesthetic-induced paradoxical excitation.

### Dynamics Around the Time of Loss of Movement

PFC–CT and bilateral PFC–PFC LFP beta/low gamma coherence was elevated prior to LOM but abruptly decreased at the time of LOM. The coincidence of LOM with a change in PFC–CT dynamics is consistent with the notion that CT plays an important role in determining arousal state under general anesthesia. Earlier studies supporting this notion found that electrophysiological changes in CT precede those in cortex during transitions to propofol-induced unconsciousness (Baker et al., 2014), and that activating the CT restores consciousness in anesthetized rodents (Alkire et al., 2009; Lioudyno et al., 2013).

PC–PFC and bilateral PFC–PFC LFP slow-delta coherence increased shortly after the decrease in PFC–CT beta/low gamma coherence coincident with LOM. We speculate that decreased beta/low gamma coherence between PFC and CT LFPs represents a functional disconnection between the PFC and CT that contributes to behavioral arrest and the appearance of cortical slow-delta oscillations under sevoflurane. A similar time course of cortico-cortical LFP power and coherence changes was observed in macaque monkeys undergoing a gradual propofol infusion (Ishizawa et al., 2016). In contexts that extend beyond anesthesia, decreased activity in subcortical arousal centers has been linked to reduced behavioral arousal and increased frontal EEG slow-delta power. Lesions of the CT in humans can lead to coma and elevated slow-delta EEG power (Schiff, 2008; Brown et al., 2010), and hyperpolarization of the thalamus is thought to contribute to cortical slow-delta oscillations during sleep (Steriade et al., 1993).

### Coherent Slow-Delta Oscillations

At doses of sevoflurane sufficient to maintain LOR, slow-delta oscillations in distant brain regions were highly coherent. This finding is consistent with the hypothesis that increasing order in neural networks may contribute to unconsciousness (Tononi, 2008; Chauvette et al., 2011; Alonso et al., 2014).

In contrast to the highly synchronous slow-delta oscillations that we observed in distant brain regions (separated by millimeters) in sevoflurane-anesthetized rats, asynchronous slow oscillations have been observed in distant regions (separated by centimeters) of the temporal cortex in propofol-anesthetized humans (Lewis et al., 2012). However, of note, between regions of human temporal cortex separated by distances on the order of those in our study (millimeters), propofol-induced slow oscillations were highly synchronous (Lewis et al., 2012). Synchronous and asynchronous oscillations may both play functional roles in anesthetic-induced unconsciousness, but within smaller or larger scale networks respectively.

### Coherent Alpha/Low Beta Oscillations

Sevoflurane and propofol are hypothesized to induce unconsciousness in mechanistically similar ways because of similar actions at GABA_A_ receptors, and similarities in the frontal EEG oscillations they induce in humans (Brown et al., 2011; Akeju et al., 2014). We therefore interpret our results in the context of a computational study investigating the mechanism for alpha oscillation generation under propofol. The study suggests that propofol-induced alpha oscillations are generated in a thalamocortical loop, and predicts that alpha coherence is high between thalamus and PFC (Ching et al., 2010). We observed an increase in PFC–CT coherence in the alpha range in rats at doses sufficient to maintain LOR, which is consistent with the hypothesis that sevoflurane-induced alpha oscillations are thalamocortically generated.

### Animals Take Distinct Emergence Paths

Emergence dynamics were variable among rats. Differing trajectories have been described in humans emerging from sevoflurane anesthesia (Chander et al., 2014; Hight et al., 2014). Also, emergence dynamics in rats were not a simple reversal of induction dynamics. Factors that may contribute to the asymmetry of induction and emergence dynamics observed in this study include the asymmetric nature of our dosing protocol and variable activation of distinct ascending arousal circuits (Kenny et al., 2016).

### Study Limitations and Future Directions

LFPs in this study were recorded with monopolar tetrodes. The smaller diameter of these electrodes likely contributes to a more local signal, while the monopolar arrangement likely contributes to a more global signal. Given that both local and distant sources likely contributed to signals, increased power in a region or coherence between regions do not necessarily reflect the generation of a signal in a region or increased communication between regions (Kajikawa and Schroeder, 2011; Lindén et al., 2011). Future studies with unit data may offer more definitive mechanistic insights.

Hudson et al. (2014) previously reported that in rats anesthetized with isoflurane, multiple brain states exist even when the inhaled dose of anesthetic is held constant. In the present study, brain state transitions were also observed at steady-state concentrations of sevoflurane, as illustrated for one animal in **Figure 2A** at 2.2% sevoflurane. Although the present study focused on characterizing the most commonly observed dynamics, future studies may aim to characterize the full range of brain states under sevoflurane anesthesia.

Identifying anesthetic-induced EEG dynamics that correlate with decreased behavioral arousal may enable better monitoring of surgical patients under general anesthesia (Purdon et al., 2013). In this study, we minimized handling of rats in order to characterize the time course of EEG and LFP dynamics induced by sevoflurane in the absence of stimulation. Further study is required to characterize dynamics accompanying behavioral endpoints that require stimulation of animals, including continuous assessment of LOR to determine the precise time at which loss of consciousness occurs (Baker et al., 2014), as well as responses to painful stimuli to assess analgesia and immobility. Finally, the asymmetric nature of our dosing protocol prevents us from considering the neurophysiology underlying neural inertia, the hysteresis of anesthetic induction and emergence (Friedman et al., 2010; Joiner et al., 2013).

In summary, sevoflurane induced frontal EEG dynamics in rats that were similar to those previously observed in humans, including increased beta/low gamma power at low doses, and increased slow-delta and alpha power at high doses. At doses sufficient to maintain LOR, rats exhibited highly coherent slow-delta oscillations. Synchronous slow-delta oscillations may contribute to sevoflurane-induced unconsciousness in rats.

## Author Contributions

JG designed and performed experiments, performed data analysis, wrote manuscript. NT designed and performed experiments, provided critical feedback on manuscript. JL performed experiments, provided critical feedback on manuscript. KV provided critical feedback on manuscript. JP provided critical feedback on manuscript. ES assisted with data analysis, provided critical feedback on manuscript. JM provided critical feedback on manuscript. EB designed experiments, provided critical feedback on manuscript. KS designed experiments, wrote manuscript.

## Conflict of Interest Statement

The authors declare that the research was conducted in the absence of any commercial or financial relationships that could be construed as a potential conflict of interest.

## Footnotes

1 http://www.calfinewire.com/datasheets/100211-tungsten9995cs.html

2 http://www.open-ephys.org/twister/

3 chronux.org
